# Interaction of cytochrome P450 3A4 with the hydrophilic ligand tetraethylene glycol

**DOI:** 10.1016/j.bbrc.2025.153040

**Published:** 2025-11-24

**Authors:** Irina F. Sevrioukova

**Affiliations:** Department of Molecular Biology and Biochemistry, University of California, Irvine, CA, 92697-3900, USA

**Keywords:** Cytochrome P450, CYP3A4, Crystal structure, Ligand binding, Imidazole, Tetraethylene glycol

## Abstract

Human cytochrome P450 3A4 (CYP3A4) is a clinically important drug-metabolizing enzyme that can oxidize a wide range of structurally diverse compounds. Due to hydrophobic nature of the active site, CYP3A4 preferably binds and biotransforms lipophilic compounds. However, small hydrophilic molecules can also interact with CYP3A4 and affect its activity via mechanisms that are incompletely understood, partly due to limited structural information. This paper describes the high-resolution X-ray structure of CYP3A4 complexed with the heme-ligating imidazole and two molecules of tetraethylene glycol (TEG1 and TEG2) originating from the crystallization solution. TEG1 binds to the active site in a curled conformation stabilized by multiple direct and water-mediated H-bonds with S119, R212, R372 and the heme propionate. TEG2, in turn, docks on the outer surface in two alternative conformations, strengthening intermolecular contacts in the crystal lattice. *In vitro* studies showed that, when bound to the active site, TEG can modulate substrate binding and functional activity of CYP3A4. Because TEG is present in polyethylene glycol mixtures widely used in food and pharmaceutical industries and has high gastrointestinal absorption, there is a possibility of *in vivo* CYP3A4-TEG complex formation that could affect intestinal drug metabolism.

## 1. Introduction

Cytochrome P450 3A4 (CYP3A4) is a highly promiscuous drug-metabolizing enzyme that primarily oxidizes lipophilic compounds [1]. This preference is defined by properties of the active site, mostly lined with hydrophobic and aromatic amino acids. However, all CYP3A4 substrates contain polar groups that contribute to binding affinity through formation of ionic and/or H-bonding interactions with polar residues present in the active site (S119, R212, T224 and several others) [2].

Hydrophilic compounds, ubiquitously present in the human environment, are not metabolized by CYP3A4 but could affect its activity. Organic solvents, such as acetonitrile, ethanol, 1-propanol, dimethyl sulfoxide, and polyethylene/polypropylene glycol [3], glutathione [4], glycerol [5], phosphate ions and di-/tricarboxylic acids [6] were shown to modulate catalytic properties of CYP3A4 by altering substrate binding, protein stability and/or interaction with the redox partner and lipid bilayer. There is structural and experimental evidence that some of these compounds can directly interact with CYP3A4. For example, in the 3nxu structure [7], dimethyl sulfoxide binds on the outer surface near the substrate channel and at the allosteric peripheral site. Glutathione modulates monooxygenase activity by inducing conformational changes in the A″-helix region which, in turn, promotes glutathione coordination to the heme [4]. Glycerol has multiple binding sites in the protein interior and can both improve protein stability and interfere with substrate metabolism [5]. Citrate, on the other hand, associates to the N-terminal region and markedly stimulates CYP3A4 activity via a complex mechanism [6]. Thus, structural insights on the binding manner of hydrophilic ligands are important for better understanding molecular mechanisms governing CYP3A4 function but remain very limited. Among ~130 crystal structures of CYP3A4 deposited to the Protein Data Bank (PDB), only four entries contain protein-bound water-soluble molecules (PDB codes 3nxu, 5a1p, 5g5j, and 5vcc).

Here we describe the high-resolution X-ray structure of CYP3A4 bound to hydrophilic imidazole and tetraethylene glycol (TEG; 194 Da). Neither compound is naturally present in the human body. However, the imidazole moiety is part of the amino acid histidine, neurotransmitter histamine, antifungal azoles and some other drugs. TEG, in turn, is present in polymeric polyethylene glycol (PEG) mixtures widely utilized as solvents, plasticizers, surfactants and lubricants in a variety of consumer products and medicines [8]. PEGs are also used in the food industry as flavor enhancers, stabilizers and thickeners, and as film-coating agents for supplements, with the acceptable daily intake up to 10 mg/kg for PEG 200–10000 [9]. High gastrointestinal absorption of low-molecular PEGs, reaching 50 % for PEG 200–400 [10], and high intestinal expression of CYP3A4 [11] raise the possibility of *in vivo* CYP3A4 exposure to TEG. This could have implications for drug metabolism because, as our structural and experimental data indicate, TEG can bind to the CYP3A4 active site and modulate substrate binding and oxidation.

## 2. Experimental procedures

### Protein expression and purification –

The C-terminally 4-histidine tagged, codon-optimized wild type (WT) and C486S Δ3–22 CYP3A4 were expressed in C41 (DE3) *Escherichia coli* cells and purified as described previously [5]. P450 content was determined according to Omura and Sato [12].

### Spectral Binding Titrations –

Equilibrium titrations of CYP3A4 were conducted in a Cary 300 spectrophotometer at ambient temperature in 0.1 M phosphate, pH 7.4. Midazolam (Tocris Bioscience) and progesterone (Sigma-Aldrich) were dissolved in dimethyl sulfoxide and added to the protein solution (2 μM) in small aliquots, with the final solvent concentration <2 %. The binding parameters were determined from fittings to the plot of the absorbance change (peak-to-trough separations in the difference spectra; ΔA) vs ligand concentration. SigmaPlot software was used for nonlinear regression data fitting to the one-site binding (equation (1)) and Hill equation (equation (2)):

(1) $$\Delta A=\Delta A_{max}∙L/\left(K_{d}+L\right)$$

(2) $$\Delta A=\Delta A_{max}∙L^{n}/\left(S_{50}^{n}+L^{n}\right)$$

where $\Delta A_{max}$ is the maximum peak-to-trough absorbance change, L – ligand concentration, $K_{d}$ – spectral dissociation constant; S_50_ - concentration of substrate giving half maximal absorbance change, and n – Hill coefficient (cooperativity factor).

### Inhibitory assay –

Effect of TEG on the functional activity of CYP3A4 was assessed fluorometrically by measuring the rate of 7-benzyloxy-4-(trifluoromethyl)-coumarin (BFC; Cayman Chemical) O-debenzylation in a reconstituted system with P450 reductase. The assay was conducted at 37 °C in 0.1 M potassium phosphate buffer, pH 7.4, containing catalase and superoxide dismutase (2 units/mL each) and 0.0025 % detergent CHAPS. Various amounts of TEG were added to the mixture of 0.6 nM P450 reductase and 0.2 nM CYP3A4 and incubated for 1 min at room temperature before adding 40 μM BFC (final concentration). After additional 1 min incubation, the reaction was initiated by adding a NADPH regeneration system consisting of 10 mM glucose, glucose-6-phosphate dehydrogenase (2 units/mL) and 0.1 mM NADPH. Before measurement, the reaction mixture was incubated at 37 °C for 30 s, after which accumulation of the fluorescent product was monitored in a Hitachi F700 fluorimeter for 90 s (λ_ex_ = 404 nm; λ_em_ = 500 nm). Within this time interval, fluorescence changes were linear. The average of three measurements was used to calculate the activity and build the [% activity] vs [TEG] plot. The IC_50_ value was derived by fitting data to a four-parameter logistic equation (equation (3)):

(3) $$y=D+(\left(A-D)/(1+10^{(X-logC)∙B}\right))$$

where X is the TEG concentration, y – activity, A and D - the maximal and minimal activity values, respectively, B - a slope factor, and C - IC_50_.

### CYP3A4 crystallization and structure determination –

Crystals of C468S CYP3A4 bound to imidazole and TEG were obtained at room temperature by a sitting drop vapor diffusion method. Protein solution (60 mg/ml) in 0.1 M potassium phosphate, pH 7.4, 0.1 M NaCl, 20 % glycerol and 2 mM dithiothreitol, was mixed with an equal volume of solution E2 from Morpheus I kit (Molecular Dimensions), containing 0.03 M (each) di-, tri-, tetra- and pentaethylene glycol, 0.1 M imidazole/MES monohydrate buffer, pH 6.5, 20 % ethylene glycol, and 10 % PEG 8000. Crystals were harvested a week later and dipped into Paratone-N oil before freezing in liquid nitrogen. The X-ray diffraction data were collected at the Stanford Synchrotron Radiation Lightsource beamline 14–1. Crystal structure was solved by molecular replacement with PHASER [13] and the 5vcc structure of WT CYP3A4 as a search model. Ligands were built with eLBOW [14] and manually fit into electron density with COOT [15]. The initial model was refined with PHENIX [14] and rebuilt with COOT. Polder omit electron density maps were calculated with PHENIX. Data collection and refinement statistics are summarized in Supplemental Table S1. The atomic coordinates and structure factors were deposited to the PDB with the ID code 9yk4.

## 3. Results and discussion

We have found previously that the C486S mutation does not have structural impacts but largely improves bacterial expression of CYP3A4 [5]. The mutant preparations were highly pure and homogenic, with the Soret band to protein absorption ratio (A_417nm/280nm_) of ≥1.7 vs 1.5–1.6 for other mutants and WT CYP3A4. As a result, the C486S variant was more amenable to crystallization.

### 3.1. Crystal structure of imidazole/TEG-bound C468S CYP3A4

**Fig. 1.**
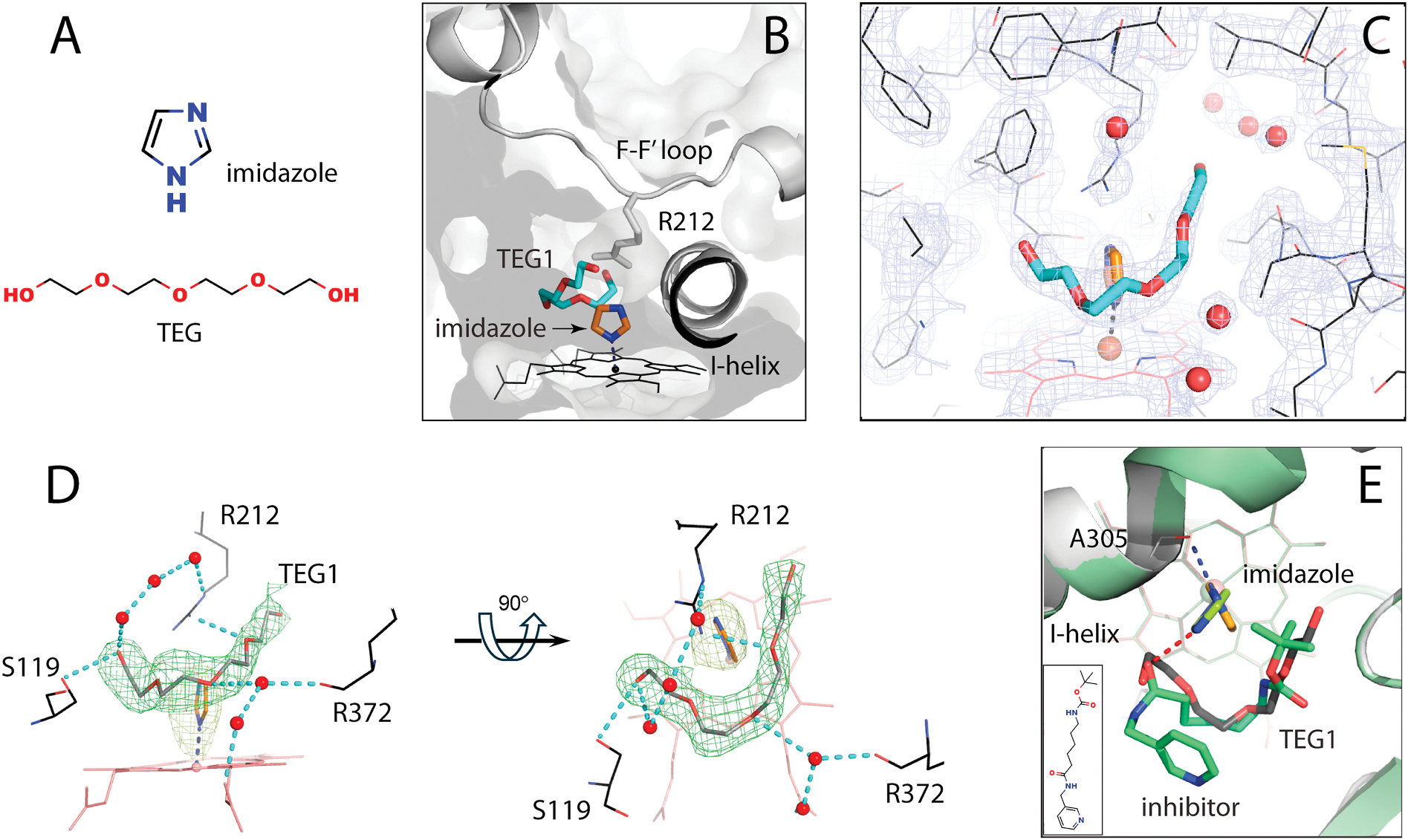
Imidazole and TEG1 bound to the active site of CYP3A4. (**A**) Chemical structures of imidazole and TEG. (**B**) Orientation of ligands (in orange and cyan sticks) relative to the I-helix and F–F′-loop. (**C**) 2F_o_-F_c_ electron density map centered at the active site and contoured at 1σ level. (**D**) Two views at the H-bonding network established by TEG1. Red balls and cyan dotted lines are water molecules and H-bonds, respectively. Green mesh is a polder omit map contoured at 3σ level. (**E**) Comparison of imidazole/TEG- and imidazole/inhibitor-bound CYP3A4 (in different shades of green; 4d6z structure). The elongated inhibitor and TEG1 occupy the same space and adopt highly similar conformations. In contrast, the imidazole molecule rotates by 70° to establish polar interactions with the inhibitor’s or A305 carbonyls (shown as red and blue dotted lines, respectively). Chemical structure of the inhibitor is shown in the *inset*.

C468S CYP3A4 was co-crystallized with imidazole and TEG unintentionally during screening trials. Both ligands originated from the crystallization solution, leading to formation of crystals with low mosaicity and high diffraction power. The structure was solved to 1.78 Ắ resolution (Table S1) and, according to the electron density maps, contained two types of ligands in the active site: a small heme-ligating molecule and an elongated one that curled around it. Based on the contents of the crystallization solution, the ligands were identified as imidazole and TEG, a flexible polymer consisting of four ethylene glycol units linked by ether bonds (Fig. 1A). A bulk of positive density was also observed on the outer surface, where TEG could fit best as well.

In the active site, imidazole ligates to the heme via the nitrogen atom, with a 2.2 Å Fe–N distance and 85° incline angle (Fig. 1B and C). The deviation from perpendicularity is caused by the proximity of TEG1 (<3.3 Å away), which curls around imidazole along the wall opposite to the central I-helix. The curled conformation is stabilized by direct and water-bridged H-bonds established by the TEG1 hydroxyl group and central O3 atom with the main/side chains of S119, R212, and R372 and the heme propionate (Fig. 1D). The other hydroxyl end-group does not engage in polar interactions and is less well defined.

It was of interest to compare the newly solved structure and the inhibitor-bound 4d6z model of CYP3A4 [16] where imidazole, also originating from the crystallization solution, prevented the inhibitor from ligating to the heme via the pyridine nitrogen. As seen from the structural overlay (Fig. 1E), the imidazole molecules interact with different polar groups and coordinate to the heme in distinct orientations. The inhibitor’s aliphatic chain, on the other hand, occupies the same space as TEG1, similarly bending between the B′-C and K-β_1_ loops and wrapping around imidazole. This conformation is stabilized by polar interactions with the side/main chains of S119 and A370 as well. Thus, there is a common trend in the inhibitor and TEG1 binding manner that could be used for prediction of association modes of elongated, flexible ligands bearing polar groups.

**Fig. 2.**
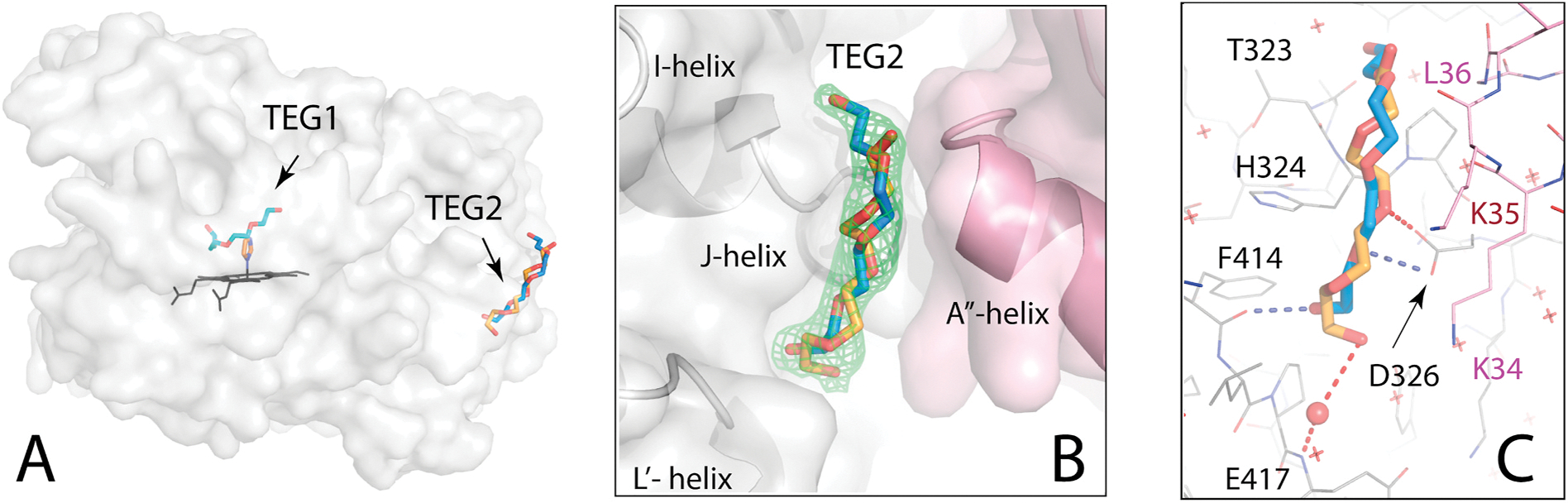
Peripheral binding of TEG2. (**A**) TEG2 binds on the outer surface in two alternative conformations (in blue and orange sticks). (**B**) The peripheral site is located at the crystallographic interface, where TEG2 interacts with the neighboring CYP3A4 molecules (in gray and pink). Green mesh is a polder omit map contoured at 3.3σ level. (**C**) Polar and van der Waals contacts established by TEG2. Only selected residues are labeled. Blue and red dotted lines are H-bonds formed by different TEG2 conformers. Red ball and crosses are water molecules.

The second TEG molecule, TEG2, binds at the crystallographic interface 25 Å away from TEG1 (Fig. 2A). Electron density examination showed that TEG2 adopts two alternative conformations and docks over the turn connecting the I- and J-helices, near the A″-helix of the symmetry related CYP3A4 molecule (Fig. 2B). The conformer with the higher occupancy (66 %; depicted in blue) H-bonds via the hydroxyl and O2 atom to the L′-helix F414 carbonyl and the J-helix D326 carboxyl, respectively (Fig. 2C). The other conformer establishes a water-mediated H-bond with the E417 main-chain amide. Via the mid portion, TEG2 forms van der Waals and long-range polar interactions with the main/side chains of K34, K35 and L36 from the A″-helix, strengthening intermolecular contacts in the crystal lattice. The fact that both the intra-cavity and peripheral sites are occupied by TEG indicates that CYP3A4 has higher affinity for and specifically selects this ligand from the PEG mixture.

Importantly, the discovery of the TEG-binding peripheral patch offers a new strategy for improvement of crystal quality and diffraction power. Crystals formed in the *I*222 space group, most common for CYP3A4, have one loosely packed molecule per asymmetric unit and are usually highly mosaic, with resolution limited to 2.4–2.8 Å even with the brightest synchrotron beams. The TEG2 binding mode suggests that crystal packing can be improved through modification of the interface, e.g. by introducing/strengthening intermolecular salt bridges or H-bonding pairs: D326-K34R, E333-T42R and/or E494-K70R. Strong polar contacts would minimize molecular motion and disorder within crystal, leading to better-quality electron density maps.

### 3.2. Effect of TEG on substrate binding and functional activity of CYP3A4

Imidazole is a well-known CYP inhibitor that upon ligation alters redox properties of the heme and slows down the flow of electrons required for substrate oxidation. For CYP3A4, the *K*_d_ and half-maximal inhibitory concentration (IC_50_) of imidazole are 3.1 mM and 1 mM, respectively [17]. PEGs and TEG, on the other hand, are considered as inert compounds and safe via oral and non-oral administration routes [8, 18,19]. To date, there are no reports on the interaction of TEG with CYP3A4 and its effects on drug metabolism. However, there is a possibility of *in vivo* CYP3A4 exposure to PEGs and, as the X-ray data showed, TEG can occupy the active site. Therefore, we investigated whether this ligand could affect substrate binding and functional activity of recombinant CYP3A4.

**Fig. 3.**
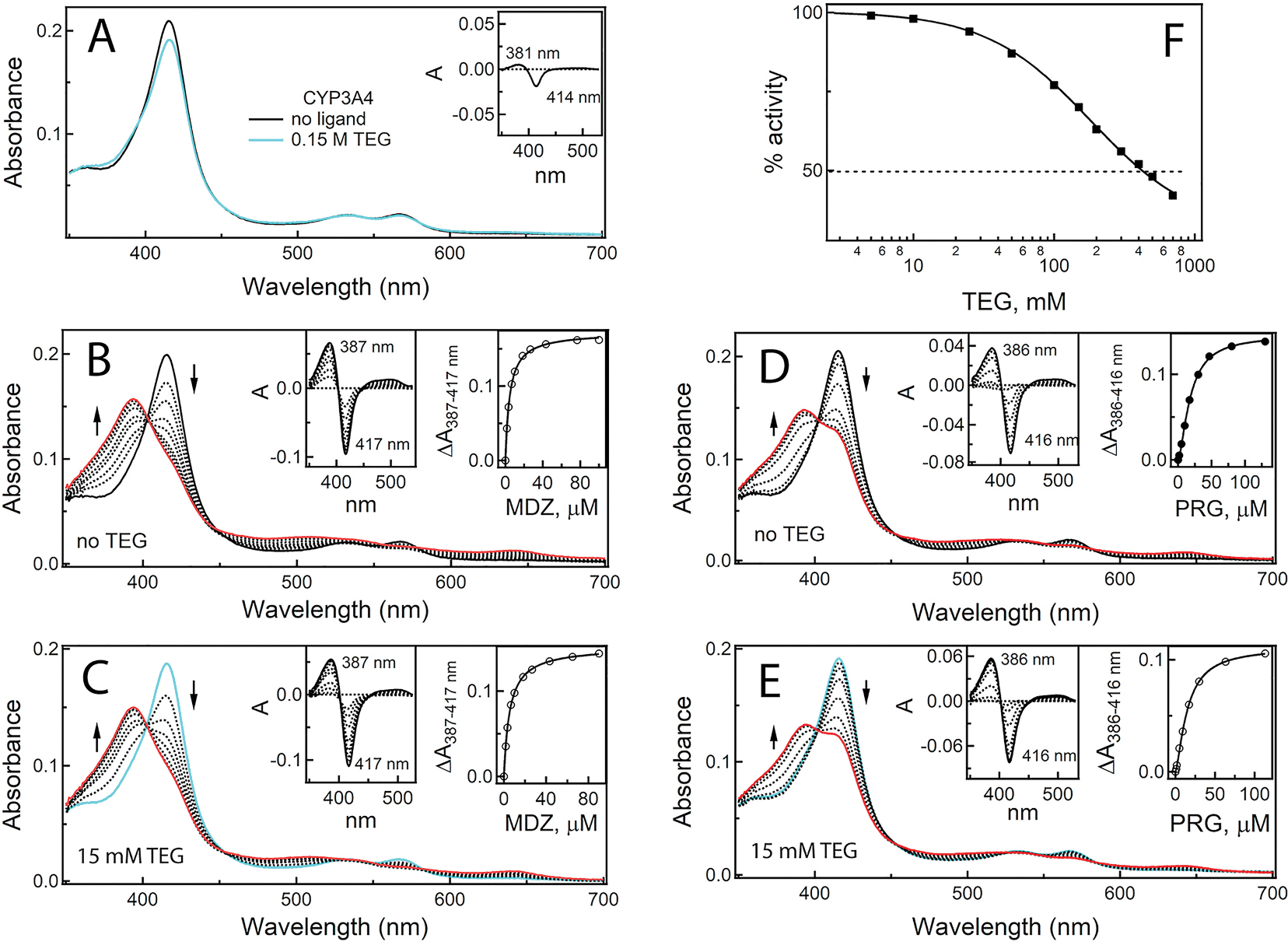
Effect of TEG on substrate binding and functional activity of CYP3A4. (**A**) TEG induces type I spectral changes in CYP3A4. The *inset* is the difference spectrum between ligand-free and TEG-bound CYP3A4. (**B**) and (**C**) Absorbance changes observed during equilibrium titration of CYP3A4 with midazolam (MDZ) in the absence and presence of 15 mM TEG, respectively. (**D**) and (**E)** Absorbance changes observed during equilibrium titration of CYP3A4 with progesterone (PRG) in the absence and presence of 15 mM TEG, respectively. In panels **B**–**E**, red spectra were recorded at the end of titrations, cyan spectra correspond to TEG-bound CYP3A4, and direction of absorbance changes is indicated by the arrows. Left and right *insets* are the difference spectra and titrations plots, respectively. Parameters derived from the hyperbolic (for MDZ) and sigmoidal fitting (for PRG) are given in Table 1. (**F**) The inhibitory plot for the BFC debenzylase activity of CYP3A4. The derived IC_50_ value for TEG is 410 mM.

It was found that, at concentration present in the crystallization solution (15 mM), TEG induces weak type I spectral changes in WT CYP3A4: a decrease in the Soret band and increase in the 380 nm area, indicative of the low-to-high spin transition in the heme iron (Fig. 3A). This confirms that TEG can enter the active site and alter the heme environment. Moreover, in the absence of imidazole, TEG seems to adopt a somewhat distinct conformation that promotes dissociation of the heme-bound water molecule, resulting in the high spin shift. Due to the small amplitude of spectral changes, it was not possible to determine spectral *K*_d_ for TEG.

Equilibrium titrations with substrates showed (Fig. 3B–E) that, in the presence of 15 mM TEG, the binding affinity of midazolam for WT CYP3A4 was decreased by 30 %, whereas the interaction with progesterone remained unaffected (Table 1). BFC does not cause spectral changes in CYP3A4 but serves as a convenient fluorogenic substrate for measuring the activity. Functional assays indicated that TEG inhibits the BFC O-debenzylation reaction with the IC_50_ of 410 mM (Fig. 3F). This finding might be biologically relevant, even though high TEG concentrations are required for significant inhibition of CYP3A4. Low-molecular PEGs have high gastrointestinal absorption [10] and are used at high concentrations (up to 60 %) [8] in formulations of drugs primarily metabolized by CYP3A4 (e.g., nifedipine, carbamazepine, diazepam, alectinib, estramustine, ibrutinib, lenvatinib, palbociclib and others). Administration of these therapeutics could increase PEG/TEG levels and promote CYP3A4-TEG interactions in the gut wall, a critical site for the first-stage drug metabolism where CYP3A4 plays the central role [20]. As a result, bioavailability/effectiveness of orally administered drugs can be altered.

In conclusion, the X-ray structure of imidazole/TEG-bound CYP3A4 provided new insights on the binding mechanism of elongated flexible ligands bearing polar groups and offered a new strategy for improvement of crystal quality. More importantly, this study showed that TEG can modulate substrate binding and catalytic ability of CYP3A4, bringing attention to possible clinical implications.

## Supplementary Material

Supplement

## Figures and Tables

**Table 1:** Effect of TEG on substrate binding to CYP3A4.

Substrate\Buffer	no TEG		15 mM TEG	
	*K*_d_ (μM)			
Midazolam	4.1 ± 0.2		5.6 ± 0.3	
	S_50_ (μM)	n_H_	S_50_ (μM)	n_H_
Progesterone	17 ± 1	1.6 ± 0.1	16 ± 2	1.5 ± 0.1

## Data Availability

All experimental data generated during this study are included in this article. Coordinates and structure factors for the X-ray model of imidazole/TEG-bound CYP3A4 are freely available at the Protein Data Bank (https://www.rcsb.org/).

## Appendix A. Supplementary data

Supplementary data to this article can be found online at https://doi.org/10.1016/j.bbrc.2025.153040.

## Abbreviations

BFC: 7-benzyloxy-4-(trifluoromethyl)-coumarin
CYP3A4: cytochrome P450 3A4
PEG: polyethylene glycol
TEG: tetraethylene glycol
