# Supplementary material for: Interaction of cytochrome P450 3A4 with the hydrophilic ligand tetraethylene glycol
Source: Biochem Biophys Res Commun. Author manuscript; Available in PMC 2026 Jan 28. (PMC12850010; doi:10.1016/j.bbrc.2025.153040)
Supplement: Supplement [file NIHMS2134216-supplement-Supplement.pdf]

SUPPLEMENTARY MATERIAL

Interaction of cytochrome P450 3A4 with the hydrophilic ligand tetraethylene glycol

Irina F. Sevrioukova

Table S1. Data collection and refinement statistics

ligand	imidazole/TEG
PDB code	9yk4
Data statistics	
Space group	I222
Unit cell parameters	a = 77 Å, b = 99 Å, c = 133 Å; α, β, γ = 90°
Resolution range (Å)	66.76 – 1.78 (1.82 – 1.78) [a]
Total reflections	199,415 (11,227)
Unique reflections	48,881 (2,768)
Redundancy	4.1 (4.1)
Completeness	99.4 (100.0)
Average $I/\sigma I$	12.1 (1.2)
R_{merge}	0.043 (0.877)
R_{pim}	0.037 (0.743)
CC ½	0.998 (0.530)
Refinement statistics	
R/R_{free} [b]	18.4/21.8
Number of atoms:	
Protein	3811
Solvent	214
R.m.s. deviations:	
Bond lengths, Å	0.007
Bond angles, °	0.889
Wilson B-factor, $Å^2$	40
Average B-factor, $Å^2$:	
Protein	53
Solvent	56
Ligands:	
TEG	55/69
imidazole	49
Ramachandran plot[c] (residues; %)	
Preferred	463 (97.7%)
Allowed	11 (2.3%)
Outliers	0

[a] Values in brackets are for the highest resolution shell.
[b] R_{free} was calculated from a subset of 5% of the data that were excluded during refinement.
[c] Analyzed with PROCHECK.
